# Children’s Exposure to Volatile Organic Compounds as Determined by Longitudinal Measurements in Blood

**DOI:** 10.1289/ehp.7412

**Published:** 2004-11-22

**Authors:** Ken Sexton, John L. Adgate, Timothy R. Church, David L. Ashley, Larry L. Needham, Gurumurthy Ramachandran, Ann L. Fredrickson, Andrew D. Ryan

**Affiliations:** ^1^University of Texas School of Public Health, Brownsville Regional Campus, Brownsville, Texas, USA; ^2^Division of Environmental Health Sciences, School of Public Health, University of Minnesota, Minneapolis, Minnesota, USA; ^3^Division of Laboratory Sciences, National Center for Environmental Health, Centers for Disease Control and Prevention, Atlanta, Georgia, USA

**Keywords:** biomarkers, blood concentrations, children’s health, cotinine, environmental justice, environmental tobacco smoke, exposure assessment, interchild variability, intrachild variability, personal exposure, volatile organic compounds

## Abstract

Blood concentrations of 11 volatile organic compounds (VOCs) were measured up to four times over 2 years in a probability sample of more than 150 children from two poor, minority neighborhoods in Minneapolis, Minnesota. Blood levels of benzene, carbon tetrachloride, trichloroethene, and *m*-/*p*-xylene were comparable with those measured in selected adults from the Third National Health and Nutrition Examination Survey (NHANES III), whereas concentrations of ethylbenzene, tetrachloroethylene, toluene, 1,1,1-trichloroethane, and *o*-xylene were two or more times lower in the children. Blood levels of styrene were more than twice as high, and for about 10% of the children 1,4-dichlorobenzene levels were ≥10 times higher compared with NHANES III subjects. We observed strong statistical associations between numerous pairwise combinations of individual VOCs in blood (e.g., benzene and *m*-/*p*-xylene, *m*-/*p*-xylene and *o*-xylene, 1,1,1-trichloroethane and *m*-/*p*-xylene, and 1,1,1-trichloroethane and trichloroethene). Between-child variability was higher than within-child variability for 1,4-dichlorobenzene and tetrachloroethylene. Between- and within-child variability were approximately the same for ethylbenzene and 1,1,1-trichloroethane, and between-child was lower than within-child variability for the other seven compounds. Two-day, integrated personal air measurements explained almost 79% of the variance in blood levels for 1,4-dichlorobenzene and approximately 20% for tetrachloroethylene, toluene, *m*-/*p*-xylene, and *o*-xylene. Personal air measurements explained much less of the variance (between 0.5 and 8%) for trichloroethene, styrene, benzene, and ethylbenzene. We observed no significant statistical associations between total urinary cotinine (a biomarker for exposure to environmental tobacco smoke) and blood VOC concentrations. For siblings living in the same household, we found strong statistical associations between measured blood VOC concentrations.

Volatile organic compounds (VOCs), many of which exhibit acute and chronic toxicity in people, are common constituents of cleaning and degreasing agents, deodorizers, dry-cleaning processes, paints, pesticides, personal care products, and solvents. Numerous VOCs are also components of automotive exhaust, industrial emissions, and environmental tobacco smoke (ETS), and they can be released into the air during showering or bathing in chlorinated water. Airborne VOCs are therefore ubiquitous in urban and nonurban environments, in indoor and outdoor settings, and in occupational and nonoccupational situations (Adgate et al. 2004a, 2004b; Edwards et al. 2001b; Kim et al. 2002; Sexton et al. 2004a, 2004b, 2004c; Wallace et al. 1985, 1987, 1988).

Although data on nonoccupational exposures to VOCs are scarce, it is apparent that concentrations of many VOCs tend to be higher indoors than outdoors and that personal (breathing zone) exposures are likely to be higher than matched in-home concentrations (Adgate et al. 2004a, 2004b; Edwards et al. 2001b; Kim et al. 2002; Sexton et al. 2004b, 2004c; Wallace et al. 1985, 1987, 1988). Research also demonstrates that nonoccupational exposures can produce corresponding blood VOC concentrations in the parts-per-trillion to parts-per-billion range (Ashley et al. 1992, 1994, 1996, 1997; Brugnone et al. 1989, 1992, 1995; Churchill et al. 2001). Children are a potentially at-risk population because they may be both more exposed to VOCs and more susceptible to adverse effects than adults. It is well established, for example, that children can be affected by different sources, pathways, and routes of exposure than adults; that children often have greater intake of air, food, beverages, soil, and dust per unit body weight and surface area; and that children differ from adults in terms of important pharmacokinetic and pharmacodymanic parameters (Aprea et al. 2000; Bearer 1995; Guzelian et al. 1992; Needham and Sexton 2000). Yet despite these concerns, it is difficult to estimate VOC-related health effects accurately because there is a paucity of information on childhood VOC exposures (Adgate et al. 2004a, 2004b; Morello-Frosch et al. 2000; Sexton et al. 2004a; Wallace 2001; Woodruff et al. 1998). In this study, we examined longitudinal measurements of blood VOC concentrations for a probability sample of elementary school–age children from two economically disadvantaged neighborhoods in Minneapolis and explored correlations with matched measurements of personal exposure to airborne VOCs and total urinary cotinine levels.

## Materials and Methods

The School Health Initiative: Environment, Learning, Disease (SHIELD) study examined children’s exposure over time to complex mixtures of environmental agents, including VOCs, ETS, metals, pesticides, and allergens.

### Subjects.

The children and families participating in the SHIELD study were from two of the most disadvantaged and ethnically diverse neighborhoods in Minneapolis: Lyndale and Whittier. For the 150 children/families in the study, total annual household income was < $9,999 for 27% of the households, between $10,000 and $19,999 for 30%, and between $20,000 and $29,999 for 21%. Just 3% of the households earned > $50,000 annually. Forty-four percent of the participating households had no occupant with a high school degree or equivalent, 32% had at least one occupant with a high school degree or equivalent, and 23% had at least one occupant who was a college graduate or technical certificate holder. In fall 1999, of the 558 children enrolled in either the Lyndale or Whittier elementary schools, 43% were African American, 20% were recent immigrants from Somalia, 20% were Hispanic (primarily Mexican American), 7% were white, 6% were Asian, and 3% were Native American. Just over half of the children (54% at Lyndale and 52% at Whittier) lived in a household where English was the primary language. As a further indicator of poverty, > 75% of the children attending each school received either free or reduced-cost meals through the National School Lunch/Breakfast Program.

### Data collection.

This study was approved by the University of Minnesota Research Subjects’ Protection Program Institutional Review Board: Human Subjects Committee. Only a brief synopsis is provided here because details of the study design (Sexton et al. 2000) and recruitment, retention, and compliance results (Sexton et al. 2003) have been published previously. A stratified random sampling strategy was used to select SHIELD participants from students in grades 2–5 (age range, 6–10 years) at either the Lyndale or Whittier elementary schools in south Minneapolis, and age-eligible siblings were also allowed to participate. In fall 1999, children and their families selected for SHIELD were contacted based on enrollment information provided by the Student Accounting Department, Minneapolis Public Schools. After successful contact, recruiters met with children and caregivers in their homes to explain the study and answer any questions. Recruiters obtained verbal and written consent/assent and administered the baseline questionnaire (which asked questions about demographic, socioeconomic, and housing attributes) to the 152 children/families who agreed to be in the study, plus 51 siblings. At enrollment the primary caregiver was asked a series of questions about smoking status and behavior, as well as questions about socioeconomic status, residential characteristics, and the child’s health.

During winter (January–February) and spring (April–May) of both 2000 and 2001, children were asked to give blood samples, which were collected at school by a trained phlebotomist. The phlebotomist attempted to obtain a 33-mL venipuncture blood sample from each child during each of the four monitoring sessions. Urine samples were also collected at the same time.

For the 2 days preceding collection of a blood sample, children, with the help of care-givers, interviews/translators, and field technicians, were asked to maintain a time–activity log, which recorded the location and approximate time they spent in seven different micro-environments. They also were asked to answer questions about the location and approximate time they spent in the presence of an active smoker. During winter and spring 2000, children also were asked to wear or carry a small passive sampler throughout the same 2-day period to measure airborne VOC concentrations. At times when it was impractical to wear or carry the monitor, such as while sleeping, children/families were instructed to place the monitor as near as possible to the child’s head (e.g., on a nightstand next to the bed). For year 1 of SHIELD, the enrollment rate was 57%, the retention rate was 85%, and > 80% of children provided requested blood and urine samples.

### Laboratory analyses.

Determination of selected VOCs in whole blood was performed by the Division of Laboratory Science, National Center for Environmental Health, Centers for Disease Control and Prevention (Atlanta, GA), using an established gas chromatography/mass spectrometry method (Ashley et al. 1992). The analytical limit of detection (nanograms per milliliter) for individual compounds was 0.010 for benzene, 0.005 for carbon tetrachloride, 0.040 for 1,4-dichlorobenzene, 0.031 for ethyl-benzene, 0.008 for styrene, 0.022 for tetra-chloroethylene, 0.016 for toluene, 0.010 for trichloroethene, 0.024 for 1,1,1-trichloroethane, 0.020 for *m*-/*p*-xylene, and 0.050 for *o*-xylene. Quality control was established by using two separate quality control materials, of which at least one was analyzed daily. Blood levels for the control pools were compared with previously established 99% confidence limits. Among the additional data validity checks were examination of gas chromatography retention time, analyte accurate mass, and instrument sensitivity, as well as comparison of mass ratios with known standards.

We obtained airborne VOC concentrations (48-hr integrated samples) with 3M model 3500 organic vapor monitors (3M Corporation, St. Paul, MN), which are charcoal-based passive air samplers. Evidence of the suitability of these monitors for personal air sampling, as well as determination of extraction efficiencies and calculation of method detection limits, has been published previously (Chung et al. 1999a, 1999b). Laboratory measurements of individual VOCs were done by T.H. Stock and M.T. Morandi at the University of Texas School of Public Health. The extraction solvent consisted of 2:1 vol:vol mix of acetone and carbon disulfide, which provided a low background for target analytes. All extracts were analyzed by gas chromatography/ mass spectrometry. Analytical and internal standards were prepared, and VOC concentrations were calculated as described previously (Chung et al. 1999b).

Total cotinine in urine samples was measured by gas chromatography–mass spectrometry in the laboratory of S.S. Hecht at the University of Minnesota, as described in previous publications (Hecht et al. 1993, 2001).

### Statistical analysis and related considerations.

Index children were sampled with selection probabilities designed to equally represent strata defined by school, grade, English-speaking versus non-English-speaking homes, and sex. Analyses were weighted to account for selection and response probabilities. Race/ethnicity was further broken down for analysis, and groups with fewer than 15 children were aggregated into a category designated “other.” Statistical analyses were performed using SAS (version 8.0; SAS Institute, Cary, NC) and S-Plus (S-Plus 2001; Insightful Corp., Seattle, WA). Analyses were performed on log-transformed laboratory values to normalize the distributions and to equalize variances, and transformed means were exponentiated to obtain geometric means. Concentrations below analytical detection limits that produced a laboratory value > 0 were included in the analyses.

We analyzed the effects of study design variables and personal exposure factors (from the time–activity logs) on blood VOC concentrations using weighted linear regression models, which included variables for season (spring compared with winter), school (Lyndale compared with Whittier), sex (male compared with female), race/ethnicity [African American, Somali immigrant, Hispanic, and Southeast Asian compared with white/Native American (“other”)], and VOC source variables (travel: ≥ 1.5 hr in a motorized vehicle over 48 hr vs. < 1.5 hr; cleaners: > 0 hr using cleaning supplies over 48 hr vs. 0 hr; cigarettes: > 0 hr spent in close proximity to a smoker over 48 hr vs. 0 hr; room deodorizers: > 0 hr using deodorizers over the past 6 months vs. 0 hr; ventilation: > 0 hr doors and/or windows were open for ventilation over 48 hr vs. 0 hr). Two-way interactions between design, source, and ventilation variables were also tested, and only significant associations are reported. This modeling of blood VOC concentrations used only results from the year 2000 because data from the time–activity logs were available only during this time.

To estimate within-child and between-child variability, blood VOC concentrations were log-transformed to make the variances homogeneous across different levels of exposure. The geometric mean for the population was designated μ, and a components-of-variance analysis was used to estimate *a*) the overall mean of the log-transformed values, log(μ); *b*) the between-child variance of log-transformed child-specific mean values, σ_P_; and *c*) the within-child variance of log-transformed levels, σ_I_. Assuming a normal distribution of individual log-transformed measurements, 95% tolerance limits (limits within which 95% of the measurements would be expected to fall) for the log-transformed values would be between *x* ± 1.96σ_I_ for a child with a mean log-concentration level of *x*. To translate the results to actual concentrations, rather than simply presenting the results in the log-transformed scale, we back-transformed these values to give corresponding intervals in the original concentration scale (nanograms per milliliter). Results in the log scale are interpreted as relative changes in concentration, so intervals in the log scale cannot be directly translated to a fixed interval in the concentration scale. Thus, we give intervals for selected individuals based on whether their mean level is an average concentration or at one or the other extreme of the distribution. Analogously, assuming they are also approximately normal, 95% tolerance limits for the distribution of mean log-transformed values among all children were computed as log(μ) ± 1.96σ_P_ and similarly back-transformed.

## Results

**Figure 1:**
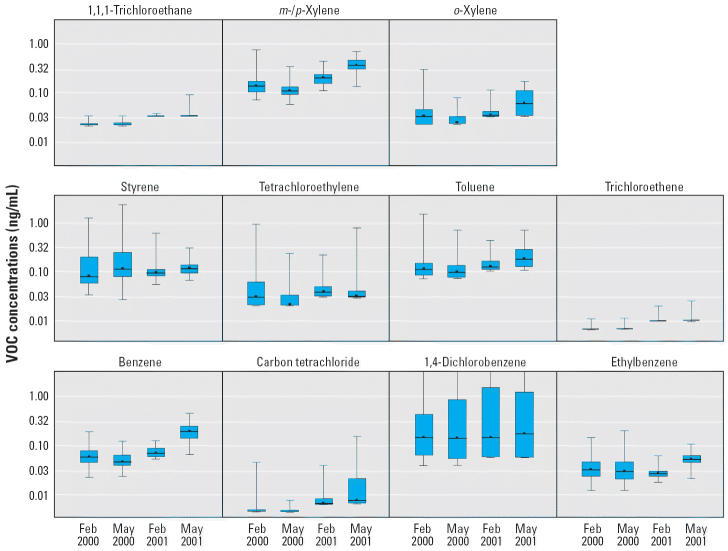
Box and whisker plots of blood VOC concentrations (ng/mL) measured in SHIELD children. Each box and whisker plot shows the median and the interquartile range (25th–75th percentile; box) and the minimum and maximum concentrations (whiskers) at a specific sampling session.

Over the 2-year, four–monitoring-session study, 134 index (randomly selected) children provided 416 blood samples. Sixty-nine children provided 4 samples, 18 provided 3 samples, 39 provided 2 samples, and 8 provided 1 sample. The number of valid samples varied by VOC and by monitoring session for two reasons. First, some samples were deemed invalid by the laboratory because of condition (e.g., clotting), failure to meet acceptability standards (e.g., insufficient blood), instrument problems, or failure of quality control parameters to be within acceptable limits. Second, the number of children providing samples changed from session to session. The distributions of blood concentrations for 11 VOCs measured during each of the four monitoring periods are summarized numerically in Table 1 and displayed graphically in Figure 1 using box and whisker plots.

During all four monitoring sessions > 50% of the samples were above the detection limit for benzene (66–97%), ethylbenzene (61–99%), styrene (57–99%), and *m*-/*p*-xylene (66–99%), whereas > 30% were above the detection limit for 1,4-dichlorobenzene (41–89%), tetrachloroethylene (37–63%), toluene (45–75%), and *o*-xylene (32–73%). The percentage of samples above the detection limit was substantially less for carbon tetrachloride (5–23%), trichloroethene (3–7%), and 1,1,1-trichloroethane (0–2%), although the percentage above zero was considerably higher (carbon tetrachloride > 38%, trichloroethene > 62%, 1,1,1-trichloroethane > 66%). Distributions of blood VOC concentrations were relatively stable over the four monitoring sessions, although median values for benzene, toluene, *m*-/*p*-xylene, and *o*-xylene were comparatively higher in May 2001. Also, in both February and May 2000, 99th-percentile values for 1,4-dichlorobenzene and styrene were comparatively higher, whereas 99th-percentile values for tetrachloroethylene and *o*-xylene were comparatively higher in February 2000.

**Figure 2:**
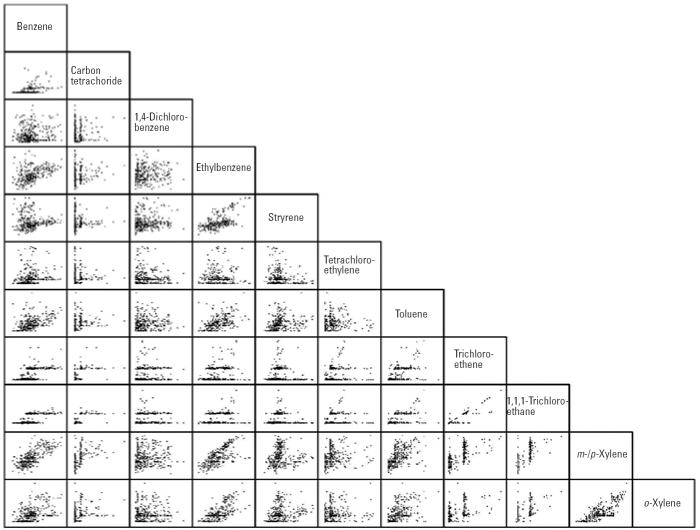
Scatterplot matrix showing relationships between blood concentrations (log_10_ ng/mL) for all pairwise combinations of individual VOCs over all sampling sessions.

Relationships between all 55 pairwise combinations of individual VOC concentrations are portrayed in Figure 2 on a log scale using a scatterplot matrix. Matched data from all four monitoring sessions are included, and the matched number of samples varies from 261 for carbon tetrachloride and ethylbenzene to 378 for *m*-/*p*-xylene and *o*-xylene. Note that the data indicate a shift in analytical detection limits over the course of the 2-year study for three VOCs (carbon tetrachloride, trichloroethene, 1,1,1-trichloroethane), which tended to be at or near the limit of detection. Results indicate that significant correlations existed between many of the pairwise combinations. Adjusted *R*^2^ values were greater than 0.50 for four pairwise combinations [1,1,1-trichloroethane and *m*-/*p*-xylene (0.52), benzene and *m*-/*p*-xylene (0.55), *m*-/*p*-xylene and *o*-xylene (0.67), and trichloroethene and 1,1,1-trichloroethane (0.84)], and between 0.40 and 0.50 for five others [1,1,1-trichloroethane and carbon tetrachloride (0.42), benzene and 1,1,1-trichloroethane (0.43), trichloroethene and *m*-/*p*-xylene (0.46), *m*-/*p*-xylene and ethylbenzene (0.46), and *o*-xylene and ethylbenzene (0.47)]. Twelve pairwise combinations had adjusted *R* ^2^ values between 0.20 and 0.40, and four were between 0.10 and 0.20. Adjusted *R*^2^ values were less than 0.05 for all 30 of the remaining 55 pairwise combinations.

The results of the components-of-variance analysis for the 11 blood VOCs measured in this study are summarized in Table 2. For each VOC, we first provide an estimate of the overall population geometric mean (column 2) and associated population 95% tolerance limits (columns 3 and 4). Next, to illustrate the spread of within-child variance, we estimate individual 95% tolerance limits for a child with a mean blood VOC level *a*) at the lower 95% tolerance limit (*L*_P_) for the overall population (columns 5 and 6), *b*) at the geometric mean (μ) for the overall population (columns 7 and 8), and *c*) at the upper 95% tolerance limit (*U*_P_) for the overall population (columns 9 and 10).

The overall population 95% tolerance interval (columns 3 and 4) provides a measure of between-child variability. For 8 of 11 compounds, the ratio of *U*_P_ to *L*_P_ ranged from 2.2 (trichloroethene) to 7.3, whereas it exceeded 10 for 1,4-dichlorobenzene (> 56,000 because a few children had elevated values), tetrachloroethylene (28.8), and styrene (16.7). Similarly, within-child variability can be estimated using the tolerance interval (columns 7 and 8) for a child with a blood level equal to the population mean [or equal to the lower or upper population 95% confidence interval (CI)]. The ratio of the *U*_μ_ to *L*_μ_ ranged from 2.5 (1,1,1-trichloroethane) to 9.8 (*m*-/*p*-xylene) for 7 of 11 compounds, and exceeded 10 for 1,4-dichlorobenzene (130), styrene (30), tetrachloroethylene (14), and benzene (14). The within-child variance can also be examined by comparing the individual 95% CI (*L*_IL_ − *U*_IL_) for a child with a mean blood concentration at the lower population 95% CI (columns 5 and 6) with the individual 95% CI (*L*_IU_ − *U*_IU_) for a child with a mean blood concentration at the upper population 95% CI (columns 9 and 10). For 9 of 11 VOCs, all except 1,4-dichlorobenzene and tetrachloroethylene, these individual 95% tolerance intervals overlap.

The ratio of (*U*_P_ − *L*_P_):(*U*_μ_ − *L*_μ_) provides a comparison of the between-child and within-child variability, where a ratio > 1 indicates between > within and a ratio < 1 indicates between < within. The between-child variability exceeded the within-child variability for 1,4-dichlorobenzene (ratio = 434) and tetra-chloroethylene (ratio = 2), and it was approximately the same (ratio ~ 1) for ethylbenzene and 1,1,1-trichloroethane. Within-child variability exceeded between-child variability for benzene, carbon tetrachloride, styrene, toluene, trichloroethene, *m*-/*p*-xylene, and *o*-xylene.

In addition to index (randomly selected) children, siblings were eligible to participate in the study provided they were also enrolled in grades 2–5 at either the Lyndale or Whittier elementary schools. Thirty-five households had an index child plus one sibling, and four households had an index child plus two siblings, for which matched blood VOC samples were available. A single matched-blood VOC sample was obtained from the index child and the sibling(s) in 7 households, two matched samples in 11 households, three in 14 households, and four in 7 households, for a total of 109 matched index–sibling blood samples. We observed moderately strong statistical associations between measured VOC concentrations in index children and their siblings for all 11 individual compounds: benzene, *R*^2^ = 0.54; carbon tetrachloride, *R*^2^ = 0.48; 1,4-dichlorobenzene, *R*^2^ = 0.82; ethylbenzene, *R* ^2^ = 0.32; styrene, *R* ^2^ = 0.69; tetrachloroethylene, *R*^2^ = 0.43; toluene, *R*^2^ = 0.56; 1,1,1-trichloroethane, *R*^2^ = 0.37; trichloroethene, *R* ^2^ = 0.44; *m*-/*p*-xylene, *R* ^2^ = 0.69; and *o*-xylene, *R*^2^ = 0.51.

Total urinary cotinine, a well-established biomarker for exposure to ETS, was measured in the children’s urine during both monitoring sessions in 2000, and results have been published previously (Hecht et al. 2001; Sexton et al. 2004a). Because exposure to ETS is a possible source of blood VOCs in nonsmokers (Ashley et al. 1996, Churchill et al. 2001), we examined the relationship between matched (within-index child) total urinary cotinine levels and concentrations of individual VOCs in blood. The total number of matched pairs ranged from 75 for ethyl-benzene to 86 for *m*-/*p*-xylene. Results indicated a lack of statistical association between cotinine and all 11 individual VOCs, with adjusted *R*^2^ values ranging from 0.0001 for *o*-xylene to 0.05 for 1,1,1-trichloroethane.

**Figure 3:**
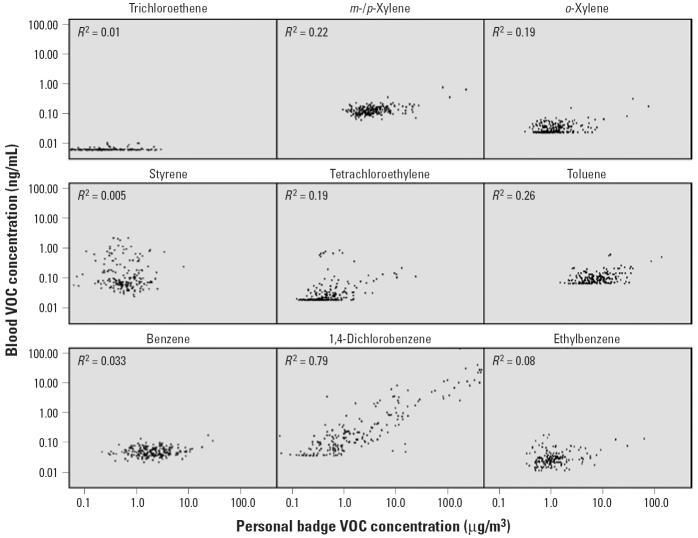
Blood VOC concentrations versus personal exposure concentrations.

During winter and spring 2000, the children wore a small, charcoal-based passive air sampler for the 2 days preceding collection of blood samples (*n* = 93 in winter 2000, *n* = 88 in spring 2000). Measurements provide an estimate of the child’s 2-day, integrated, personal exposure (across all indoor and outdoor microenvironments) to airborne VOCs. The relationships between matched (within-index child) personal VOC exposures and blood VOC concentrations are shown in Figure 3. There was a strong statistical association for 1,4-dichlorobenzene (*R*^2^ = 0.79) and a moderate association for *m*-/*p*-xylene (*R*^2^ = 0.22), *o*-xylene (*R* ^2^ = 0.19), tetrachloroethylene (*R*^2^ = 0.19), and toluene (*R*^2^ = 0.26). Little or no statistical association was observed for trichloroethene (*R* ^2^ = 0.01), styrene (*R* ^2^ = 0.005), benzene (*R*^2^ = 0.033), or ethylbenzene (*R*^2^ = 0.08).

**Figure 4:**
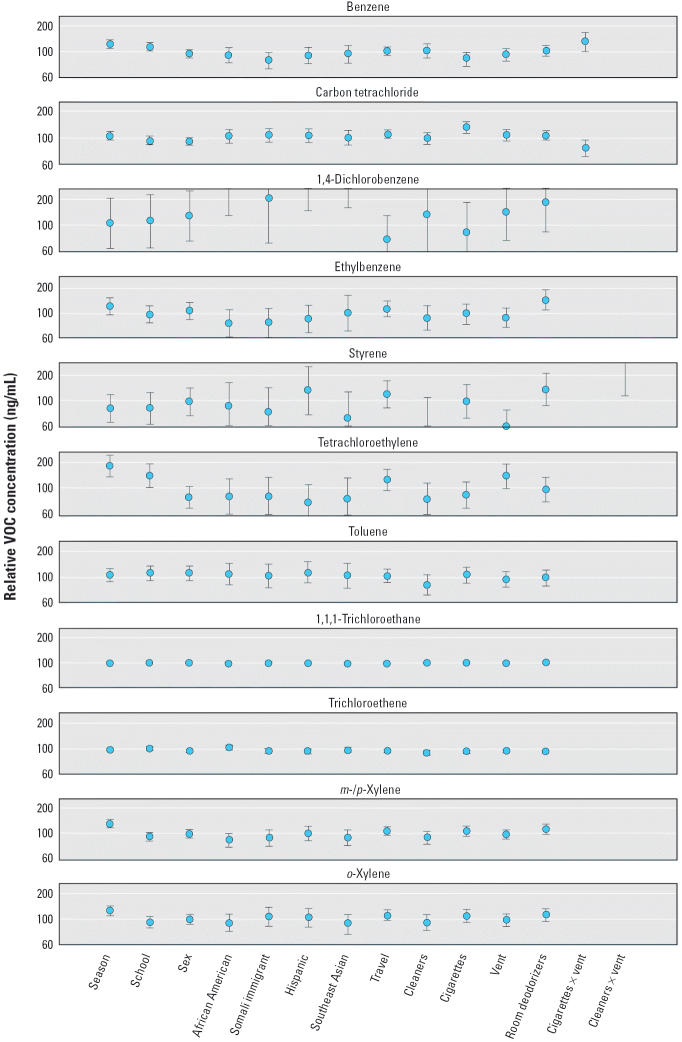
Estimated mean relative blood VOC concentration for each variable in the regression model. Vent, ventilation. Each point represents the estimated mean effect and the deviation from the mean using the 95% CI (calculated as the anti-log of the parameter estimates from each regression model). Season, winter 2000 versus spring 2000; school, Whittier versus Lyndale; sex, female versus male; African American versus other (including whites); Somali immigrant versus other (including whites); Hispanic versus other (including whites); Southeast Asian versus other (including whites); travel, > 1.5 hr on highway or road today; cleaners, > 0 hr spent using cleaning supplies today; cigarettes, > 0 cigarettes smoking in your presence today; vent, > 0 hr door and windows open for ventilation today; cigarettes × vent; cleaners × vent.

Each data point in Figure 4 represents the estimated main effect of the variable or two-way interaction compared with the designated referent category in terms of relative VOC concentration (nanograms per milliliter). The 100% line indicates that blood VOC concentrations are approximately the same relative to the referent value—suggesting that there is no discernible effect on blood VOC concentrations. The variation about the mean is represented by 95% CI, which is calculated from the standard error of the parameter estimate from each regression model. Results were considered to be statistically significant when the CI did not include 100%. For example, the model indicates that mean blood benzene levels in spring 2000 were 22% higher than winter 2000 levels, and because the CI does not include the 100% line, this result is considered significant.

Results suggest that mean blood concentrations were significantly higher in spring than winter 2000 for benzene (22% higher), tetrachloroethylene (77%), *m*-/*p*-xylene (27%), and *o*-xylene (25%). Blood VOC concentrations were similar for children enrolled at the Whittier and Lyndale schools, except for benzene (14%), tetrachloroethylene (37%), and trichloroethene (7%), which were higher in children attending Lyndale. We observed no significant differences in blood VOC concentrations between males and females, but mean levels of 1,4-dichlorobenzene were 262% higher in African-American, 310% higher in Hispanic, 97% higher in Somali immigrant, and 419% higher in Southeast-Asian children compared with a group designated “other,” which included white and Native American children. Ethylbenzene concentrations in blood were 34% higher for children whose caregiver reported using home deodorizers during the 6 months preceding the study. Although benzene blood concentrations were not significantly increased by smokers in the home and were slightly decreased by ventilation, ventilation in homes with smokers was associated with 34% higher levels than would be expected by the product of the two effects. Conversely, for carbon tetrachloride there was a 30% increase in blood concentrations for children from homes with smokers, but the interaction effect made concentrations 24% lower in children who reported both exposure to ETS and windows or doors open for ventilation than would be expected by the product of the two effects. Styrene levels in blood were significantly lower (50%) for ventilated homes but were 284% higher than expected in children living in homes where cleaners were used and windows or doors were also open for ventilation. As always, one must interpret these with results with caution because of the issues raised by multiple comparison.

## Discussion

Several studies have shown that internal doses of some VOCs, including benzene, styrene, and toluene, are elevated in smokers (Ashley et al. 1996; Churchill et al. 2001; Wallace et al. 1987). For nonsmokers, exposure to VOCs can be elevated in a variety of ways, including carrying out routine cooking and cleaning activities, being in close proximity to a smoker, riding inside a car in heavy traffic, refueling a vehicle, conducting hobby-related activities indoors, coming into contact with dry-cleaning processes or products, using cosmetics, and applying paints, paint thinners, furniture strippers, stains, and varnishes (Adgate et al. 2004a, 2004b; Ashley et al. 1992, 1994, 1996; Edwards et al. 2001a, 2001b; Kim et al. 2002; Sexton et al. 2004a, 2004b, 2004c; Wallace et al. 1985, 1987, 1988). Overall, available studies indicate that blood VOC levels are in the parts-per-trillion to parts-per-billion range for most people with no known occupational exposure, and that smoking is the largest confounder in discerning the influence of other environmental exposures (Ashley et al. 1996; Brugnone et al. 1989; Churchill et al. 2001; Wallace et al. 1987).

The internal doses that result from environmental exposures to VOCs are a function of complicated biologic, chemical, and physical processes. The evidence on the pharmacokinetics of VOCs suggests that a series of dynamic mechanisms control the uptake, deposition in body stores, metabolism, and elimination of these chemicals. Most of the internal dose of VOCs is eliminated in a matter of hours. However, a portion is removed over a much longer time period, and it is possible that VOCs may bioaccumulate with repeated exposures of sufficient duration. The half-life of VOCs in blood is short (hours), intermediate (days) in muscle tissue, and longer (months, years) in adipose tissue. The fraction of deposition at different sites in the body depends on two key factors: the length of exposure and the lipid solubility of the VOC (Ashley et al. 1996; Ashley and Prah 1997).

None of the children in this study were active smokers, nor were any of the children exposed in an occupational setting. Their VOC exposures and related blood levels are the product of concentrations in the air, water, soil, dust, food, beverages, and consumer products with which they came into contact through everyday activities and behaviors. Data from the time–activity logs indicate that in winter and spring 2000 the children spent most of their time indoors at home or at school and that they had relatively little exposure to ETS. On average, the children spent 65% (SD = 6.6) of each day inside at home, 25% (SD = 4.4) inside at school, 3.2% (SD = 5.4) inside in other locations, 1.2% (SD = 2.0) outside at home, 1.3% (SD = 1.0) outside at school, 0.7% (SD = 0.7) outside in other locations, and 3.6% (SD = 1.9) traveling in a vehicle. They were in close proximity to a smoker inside a building for an average of 1.3% (SD = 3.8) of each day and in close proximity to a smoker inside a vehicle for 0.1% (SD = 0.2) (Adgate et al. 2004a).

To put measured blood concentrations in perspective, Table 3 provides a comparison of results (arithmetic mean, median, and 95th percentile) from 134 SHIELD children between 6 and 10 years of age (one to four samples collected over 2 years), with findings from one-time measurements in more than 550 adults (≥18 years, including smokers) with no known occupational exposure who participated in the Third National Health and Nutrition Examination Survey (NHANES III) (Ashley et al. 1994). Blood concentrations of carbon tetrachloride and trichloroethene were near limits of detection in both studies. Mean and median levels of benzene and *m*-/*p*-xylene were comparable in both studies, although 95th percentile values were substantially higher in NHANES III (0.14 vs. 0.48 ng/mL for benzene and 0.32 vs. 0.78 ng/mL for *m*-/*p*-xylene). It is worth noting that for benzene and *m*-/*p*-xylene highest 95th percentile SHIELD values in specific seasons were comparable with NHANES III values: 0.40 in spring 2001 versus 0.48 ng/mL in NHANES III for benzene and 0.60 in spring 2001 versus 0.78 ng/mL in NHANES III for *m*-/*p*-xylene. Mean, median, and 95th percentile concentrations were two or more times higher in NHANES III for ethylbenzene, tetrachloroethylene, toluene, 1,1,1-trichloroethane, and *o*-xylene. Mean and 95th percentile blood levels of 1,4-dichlorobenzene and mean, median, and 95th percentile levels of styrene were more than twice as high in SHIELD children compared with NHANES III.

Because the NHANES III sample included smokers, it is not surprising that many blood VOCs were higher compared with SHIELD children. The fact that styrene concentrations were substantially higher in the children is unexpected, particularly because styrene is one of several VOCs known to be elevated in smokers’ blood (Ashley et al. 1996; Churchill et al. 2001; Wallace et al. 1987). The source of the children’s exposure to styrene is not known, and related health risks (e.g., effects on the central nervous system, liver, and red blood cells) are uncertain. Further research is needed to elucidate the sources, pathways, and routes of exposure to styrene for children in general and poor minority children in particular.

The blood concentrations of 1,4-dichloro-benzene in some SHIELD children were among the highest ever measured by the National Center for Environmental Health, Centers for Disease Control and Prevention. Thirteen of the 134 index children with at least one blood sample had 1,4-dichlorobenzene concentrations > 10 ng/mL (a total of 26 samples exceeded 10 ng/mL). For two of these children all four blood values were > 10 ng/mL, and for seven, two values were > 10 ng/mL. Although the SHIELD study was not designed to identify specific VOC sources, the evidence suggests that children were typically exposed inside their homes (*R*^2^ = 0.77 for indoor residential vs. blood concentrations). Because 1,4-dichlorobenzene is a common constituent of air fresheners and deodorizers, and because field staff reported the pervasive odor of these products in some households, we speculate that elevated blood levels in this population may be caused by frequent use of these kinds of consumer products. Additional research is needed to determine the sources and pathways for children’s exposure to 1,4-dichlorobenzene, and to better ascertain related health risks (e.g., cancer, central nervous system, respiratory system, kidney).

Because longitudinal measurements of blood VOC concentrations were made in the same children over time, the SHIELD data provide one of the first opportunities to estimate interchild and intrachild variability. For 2 of 11 VOCs (1,4-dichlorobenzene and tetrachloroethylene), between-child variability was greater than within-child variability, a condition that tends to complicate efforts to distinguish differences between individuals with a limited number of measurements and a constrained sample size. Between-child variability was less than within-child variability for seven VOCs (benzene, carbon tetrachloride, styrene, toluene, trichloroethene, *m*-/*p*-xylene, *o*-xylene) and approximately the same for ethylbenzene and 1,1,1-trichloroethane. The ratio of between-child to within-child variability is important because it can affect determinations of the minimum sample size and number of measurements needed to detect differences between groups of individuals (e.g., power calculations). In this study, children’s blood samples were drawn during the school day at convenient times. Future research should examine whether the timing of blood collection (e.g., early morning vs. end of day) has an effect on within- and between-child variability.

Because they have many common sources, numerous individual blood VOCs were highly correlated (e.g., *R*^2^ = 0.84 for trichloroethene and 1,1,1-trichloroethane, *R*^2^ = 0.67 for *m*-/*p*-xylenes and *o*-xylene, *R*^2^ = 0.55 for benzene and *m*-/*p*-xylenes, *R* ^2^ = 0.52 for 1,1,1-trichloroethane and *m*-/*p*-xylenes). Although we expected that airborne VOC levels would be the major determinant of blood VOC concentrations, 2-day, integrated personal air samples explained < 10% of the variance in blood levels for four of nine VOCs (benzene, ethylbenzene, styrene, trichloroethene) for which matched air–blood samples were available, and between 19 and 26% for four others (tetrachloroethylene, toluene, *m*-/*p*-xylenes, *o*-xylene). Personal air levels explained most of the variance in matched blood concentrations only for 1,4-dichlorobenzene (*R*^2^ = 0.79).

A previous study (Mannino et al. 1995) in adults known to be occupationally exposed to gasoline fumes and automotive exhaust found substantially higher correlations in nonsmokers between personal air measurements (5–8 hr integrated occupational samples) and blood concentrations for several VOCs (ethylbenzene, *R* = 0.82; toluene, *R* = 0.88; *m*-/*p*-xylenes, *R* = 0.94; *o*-xylene, *R* = 0.90). The relatively low correlations in SHIELD children could be explained by one or more of several possible reasons: the longer averaging time for personal air samples (48 hr vs. 5–8 hr); different exposure magnitudes, durations, and frequencies (e.g., longer-term, relatively lower community exposures for the children vs. shorter-term, relatively higher occupational exposures); differences in pharmacokinetics (e.g., absorption, deposition, metabolism, elimination) between children and adults; and the contribution of other routes of exposure (e.g., ingestion of VOCs in food or beverages, absorption through the skin during bathing or showering).

Although smoking is known to be an important determinant of blood VOC concentrations (Ashley et al. 1996; Churchill et al. 2001; Wallace et al. 1987), evidence of a link between ETS exposure and blood VOC levels in nonsmokers is scarce. We have previously reported results of total urinary cotinine measurements, a biomarker for nicotine and hence ETS exposure in nonsmokers, for SHIELD children. Findings indicated that measured concentrations in the children’s urine were comparable with other ETS studies in non-smoking adults (Hecht et al. 2001) and that concentrations varied by ethnicity/race, with highest levels observed in African-American children and lowest levels in Hispanic and Somali immigrant children (Sexton et al. 2004a). When we examined matched (within-child) measurements of total urinary cotinine and blood VOC levels in winter and spring 2000, we found virtually no correlation between ETS exposure and any of the 11 measured blood VOCs (0.0001 ≤*R* ^2^ ≤0.05), despite the fact that some children were exposed to relatively high levels of ETS (Hecht et al. 2001; Sexton et al. 2004a) that might reasonably be expected to influence blood VOC concentrations, particularly levels of benzene, styrene, and toluene (Ashley et al. 1996; Churchill et al. 2001; Wallace et al. 1987). One possible explanation for the lack of statistical association is the relatively stable levels of total urinary cotinine measured over time for each child, which meant that within-child variability was comparatively low (Sexton et al. 2004a). On the other hand, these results are consistent with relatively low correlations observed between personal air exposure and most blood VOC concentrations, which suggests that, except for 1,4-dichlorobenzene, airborne levels may not have been the dominant factor influencing children’s blood VOC concentrations.

## Conclusions

The SHIELD study is one of the first to measure, over time, blood concentrations of VOCs in a probability sample of children. Results indicate that childhood exposures to some compounds equaled or exceeded VOC exposures of adults, including smokers, in an earlier national survey, and that within-child variability was greater than between-child variability for 7 of 11 individual VOCs. Matched personal exposure (breathing zone) measurements explained ≤25% of the variance in blood concentrations for 10 of 11 compounds, whereas matched urinary cotinine measurements (an ETS exposure biomarker) explained ≤5% of the variance in blood VOC levels for each of the 11 compounds. Further research is needed to better understand the sources, pathways, and routes of children’s exposure to VOCs.

## Figures and Tables

**Table 1:** Distribution of blood VOC concentrations (ng/mL) among SHIELD children.

				Percentile						
VOC	Month/year	No.	% > DL	10th	25th	50th	75th	90th	95th	99th
Benzene	Feb 2000	112	97.4	0.03	0.04	0.06	0.07	0.08	0.09	0.16
	May 2000	113	94.0	0.03	0.04	0.04	0.06	0.07	0.08	0.10
	Feb 2001	63	66.3	0.05	0.06	0.07	0.08	0.10	0.10	0.11
	May 2001	72	80.9	0.10	0.14	0.18	0.22	0.28	0.40	0.41
Carbon tetrachloride	Feb 2000	106	5.2	0.00	0.00	0.00	0.00	0.00	0.01	0.04
	May 2000	110	9.4	0.00	0.00	0.00	0.00	0.00	0.01	0.01
	Feb 2001	60	22.1	0.01	0.01	0.01	0.01	0.01	0.02	0.02
	May 2001	34	22.5	0.01	0.01	0.01	0.01	0.02	0.03	0.14
1,4-Dichlorobenzene	Feb 2000	112	88.7	0.04	0.06	0.14	0.38	6.00	12.00	470.0
	May 2000	114	79.5	0.04	0.05	0.12	0.96	5.50	27.00	140.0
	Feb 2001	56	41.1	0.05	0.06	0.22	2.80	13.00	22.00	24.00
	May 2001	86	65.2	0.05	0.05	0.15	1.10	2.20	18.00	34.00
Ethylbenzene	Feb 2000	92	79.1	0.02	0.02	0.03	0.05	0.07	0.08	0.12
	May 2000	86	66.7	0.01	0.02	0.03	0.04	0.05	0.07	0.17
	Feb 2001	63	61.1	0.02	0.02	0.02	0.03	0.03	0.03	0.04
	May 2001	88	98.9	0.03	0.04	0.05	0.06	0.08	0.09	0.10
Styrene	Feb 2000	103	89.6	0.04	0.05	0.07	0.18	0.74	0.85	1.00
	May 2000	108	92.3	0.05	0.07	0.09	0.18	0.54	0.68	2.00
	Feb 2001	54	56.8	0.06	0.07	0.09	0.10	0.11	0.11	0.54
	May 2001	88	98.9	0.08	0.09	0.11	0.12	0.17	0.21	0.27
Tetrachloroethylene	Feb 2000	108	62.6	0.02	0.02	0.03	0.05	0.11	0.65	0.82
	May 2000	113	43.6	0.02	0.02	0.02	0.03	0.05	0.09	0.21
	Feb 2001	60	46.3	0.03	0.03	0.03	0.04	0.06	0.09	0.19
	May 2001	79	37.1	0.03	0.03	0.03	0.04	0.09	0.10	0.69
Toluene	Feb 2000	106	73.9	0.06	0.07	0.10	0.13	0.20	0.25	0.49
	May 2000	102	55.6	0.07	0.07	0.08	0.11	0.19	0.20	0.55
	Feb 2001	60	45.3	0.09	0.10	0.11	0.14	0.16	0.19	0.38
	May 2001	79	75.3	0.10	0.12	0.17	0.25	0.34	0.37	0.61
Trichloroethene	Feb 2000	100	7.0	0.01	0.01	0.01	0.01	0.01	0.01	0.01
	May 2000	115	5.1	0.01	0.01	0.01	0.01	0.01	0.01	0.01
	Feb 2001	59	3.2	0.01	0.01	0.01	0.01	0.01	0.01	0.02
	May 2001	88	6.7	0.01	0.01	0.01	0.01	0.01	0.02	0.02
1,1,1-Trichloroethane	Feb 2000	108	0.0	0.02	0.02	0.02	0.02	0.02	0.03	0.03
	May 2000	114	0.0	0.02	0.02	0.02	0.02	0.02	0.02	0.03
	Feb 2001	63	1.1	0.03	0.03	0.03	0.03	0.04	0.04	0.04
	May 2001	78	2.2	0.03	0.03	0.03	0.04	0.04	0.06	0.07
*m*-/*p*-Xylene	Feb 2000	113	98.3	0.10	0.11	0.13	0.17	0.21	0.22	0.74
	May 2000	115	98.3	0.09	0.10	0.11	0.13	0.17	0.17	0.20
	Feb 2001	63	66.3	0.15	0.16	0.19	0.23	0.31	0.31	0.32
	May 2001	88	98.9	0.23	0.30	0.37	0.47	0.57	0.60	0.66
*o*-Xylene	Feb 2000	113	73.0	0.02	0.02	0.03	0.05	0.06	0.08	0.30
	May 2000	114	44.4	0.02	0.02	0.02	0.03	0.04	0.05	0.07
	Feb 2001	63	31.6	0.03	0.03	0.03	0.04	0.05	0.06	0.11
	May 2001	88	66.3	0.03	0.04	0.07	0.11	0.13	0.14	0.16

DL, detection limit.

**Table 2:** Summary of intrachild and interchild variability for blood VOC concentrations (ng/mL).

				Intrachild tolerance interval for					
		Interchild tolerance interval for population*^b^*		Individual with mean of *L*_p_*^c^*		Individual with mean of μ*^d^*		Individual with mean of *U*_p_*^e^*	
Compound	Overall population geometric mean*^a^* (μ)	*L*_P_	*U*_P_	*L*_IL_	*U*_IL_	*L*_μ_	*U*_μ_	*L*_IU_	*U*_IU_
Benzene	0.063	0.026	0.152	0.007	0.097	0.017	0.233	0.042	0.559
Carbon tetrachloride	0.005	0.002	0.011	0.001	0.006	0.002	0.014	0.004	0.030
1,4-Dichlorobenzene	0.242	0.001	56.436	0.000	0.012	0.021	2.728	5.009	635.803
Ethylbenzene	0.033	0.012	0.087	0.004	0.035	0.012	0.092	0.031	0.244
Styrene	0.110	0.027	0.450	0.005	0.149	0.020	0.608	0.082	2.487
Tetrachloroethylene	0.033	0.006	0.173	0.002	0.024	0.009	0.127	0.045	0.661
Toluene	0.117	0.045	0.306	0.017	0.121	0.043	0.315	0.114	0.824
Trichloroethene	0.007	0.005	0.011	0.003	0.008	0.004	0.012	0.007	0.018
1,1,1-Trichloroethane	0.027	0.018	0.041	0.012	0.029	0.017	0.043	0.026	0.064
*m*-/*p*-Xylene	0.172	0.074	0.401	0.024	0.230	0.055	0.536	0.129	1.249
*o*-Xylene	0.039	0.015	0.100	0.006	0.041	0.014	0.105	0.037	0.271

a μ = 10^mean[log(^^x^^)]^, the estimated geometric mean, calculated by back-transforming the average of within-child mean log concentrations.

b (*L*_P_, *U*_P_) = 95% tolerance interval for the population of individual mean serum levels, calculated using between-child SD, σ_P_, for the log-transformed data and back-transforming: log(μ) ± 1.96σ_P_.

c (*L*_IL_, *U*_IL_) = Tolerance interval for an individual with mean serum level equal to the lower tolerance limit of the population, *L*_P_, and calculated using within-child SD, σ_I_, and back-transforming: log(*L*_P_) ± 1.96σ_I_.

d (*L*_μ_, *U*_μ_) = Tolerance interval for an individual with mean serum level equal to the estimated geometric mean of the population, μ, and calculated using within-child SD, σ_I_, and back-transforming: log(μ) ± 1.96σ_I_.

e (*L*_IU_, *U*_IU_) = Tolerance interval for an individual with mean level equal to the upper tolerance limit of the population, *U*_P_, calculated using within-child SD, σ_I_, and back-transforming: log(*U*_P_) ± 1.96σ_I_.

**Table 3:** Comparison of blood VOC concentrations (ng/mL) for SHIELD children and selected adult participants (including smokers) in NHANES III.

	Mean*^a^*		Median		95th Percentile	
Compound	SHIELD*^b^*	NHANES*^c^*	SHIELD	NHANES	SHIELD	NHANES
Benzene	0.08	0.13	0.08	0.06	0.14	0.48
Carbon tetrachloride	0.01	ND	0.01	ND	0.01	ND
1,4-Dichlorobenzene	4.22	1.9	0.21	0.33	24.5	9.2
Ethylbenzene	0.04	0.11	0.03	0.06	0.07	0.25
Styrene	0.17	0.07	0.12	0.04	0.50	0.18
Tetrachloroethylene	0.06	0.19	0.03	0.06	0.22	0.62
Toluene	0.14	0.52	0.11	0.28	0.27	1.5
Trichloroethene	0.01	0.02	0.01	ND	0.01	0.02
1,1,1-Trichloroethane	0.03	0.34	0.03	0.13	0.03	0.80
*m*-/*p*-Xylene	0.21	0.37	0.19	0.19	0.32	0.78
*o*-Xylene	0.05	0.14	0.04	0.11	0.09	0.30

ND, below limit of detection.

a Arithmetic mean.

b Participants included 134 children with at least one blood sample in 2000 or 2001 (average values were used for children with more than one blood sample).

c Between 574 and 1,037 participants, depending on the VOC (from Ashley et al. 1994).
